# Supplementary figures and images for: Genetic diversity and population structure of Uganda’s yam (Dioscorea spp.) genetic resource based on DArTseq
Source: PLoS One. 2023 Feb 14;18(2):e0277537. doi: 10.1371/journal.pone.0277537 (PMC9928066; doi:10.1371/journal.pone.0277537)

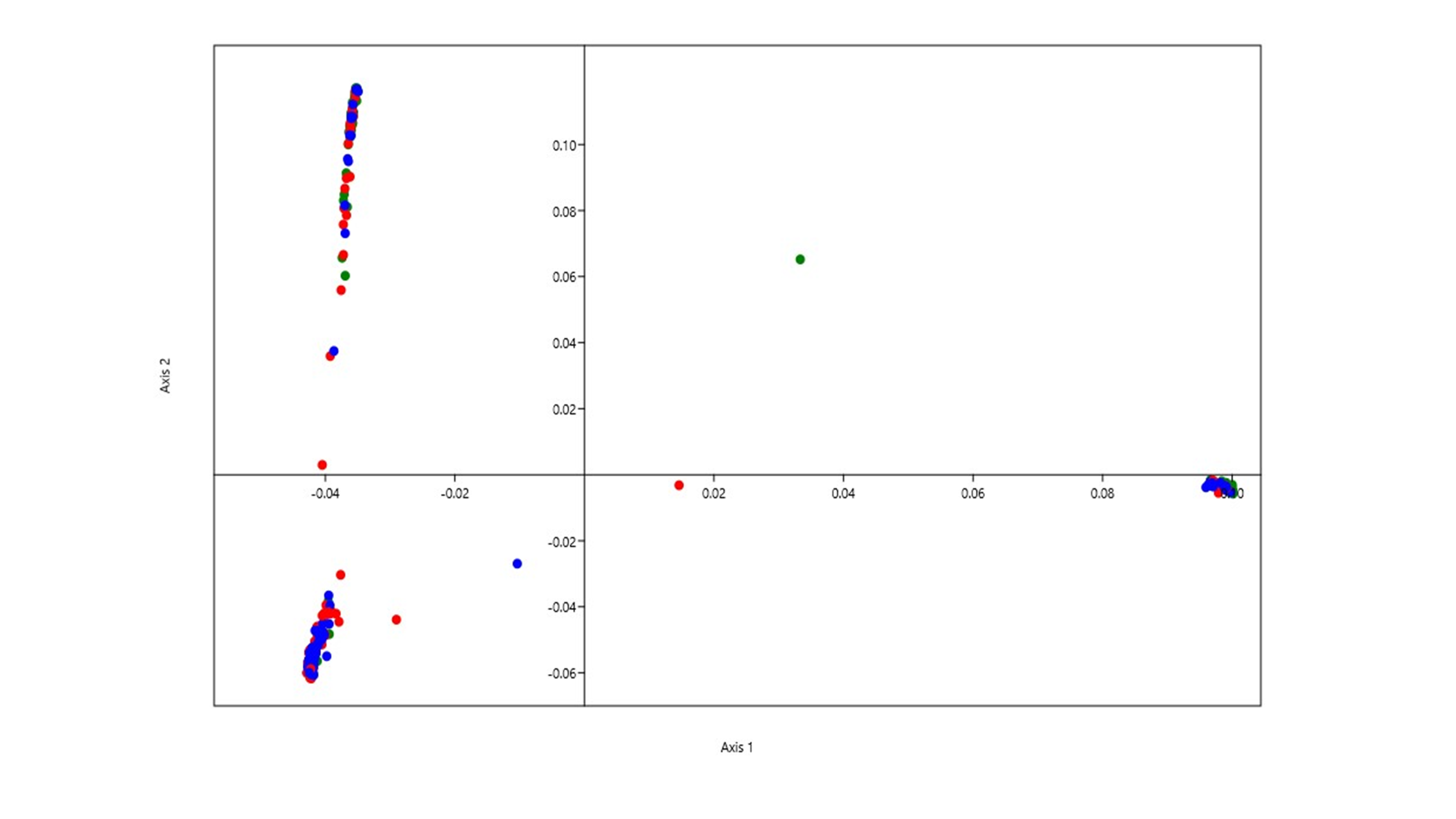

Supplement: S1 Fig — (Uganda = Green, Nigeria = blue, Ghana = Red). (TIF) [file pone.0277537.s001.tif]
