# Supplementary material for: Genetic diversity and population structure of Uganda’s yam (Dioscorea spp.) genetic resource based on DArTseq
Source: PLoS One. 2023 Feb 14;18(2):e0277537. doi: 10.1371/journal.pone.0277537 (PMC9928066; doi:10.1371/journal.pone.0277537)
Supplement: S1 Table — (DOCX) [file pone.0277537.s002.docx]

**S1Table. List of genotypes and their geographical origins**

| **No.** | **Genotype** | **Description** | **Geographical origin** | **Institution** |
| --- | --- | --- | --- | --- |
| 1 | UGY16002 | Landrace | Uganda | NARO-NaCRRI |
| 2 | UGY16003 | Landrace | Uganda | NARO-NaCRRI |
| 3 | UGY16004 | Landrace | Uganda | NARO-NaCRRI |
| 4 | UGY16006 | Landrace | Uganda | NARO-NaCRRI |
| 5 | UGY16010 | Landrace | Uganda | NARO-NaCRRI |
| 6 | UGY16011 | Landrace | Uganda | NARO-NaCRRI |
| 7 | UGY16013 | Landrace | Uganda | NARO-NaCRRI |
| 8 | UGY16016 | Landrace | Uganda | NARO-NaCRRI |
| 9 | UGY16022 | Landrace | Uganda | NARO-NaCRRI |
| 10 | UGY16026 | Landrace | Uganda | NARO-NaCRRI |
| 11 | UGY16027 | Landrace | Uganda | NARO-NaCRRI |
| 12 | UGY16029 | Landrace | Uganda | NARO-NaCRRI |
| 13 | UGY16033 | Landrace | Uganda | NARO-NaCRRI |
| 14 | UGY16035 | Landrace | Uganda | NARO-NaCRRI |
| 15 | UGY16037 | Landrace | Uganda | NARO-NaCRRI |
| 16 | UGY16039 | Landrace | Uganda | NARO-NaCRRI |
| 17 | UGY16041 | Landrace | Uganda | NARO-NaCRRI |
| 18 | UGY16042 | Landrace | Uganda | NARO-NaCRRI |
| 19 | UGY16045 | Landrace | Uganda | NARO-NaCRRI |
| 20 | UGY16064 | Clone | Uganda | NARO-NaCRRI |
| 21 | UGY16065 | Clone | Uganda | NARO-NaCRRI |
| 22 | UGY16066 | Clone | Uganda | NARO-NaCRRI |
| 23 | UGY16068 | Landrace | Uganda | NARO-NaCRRI |
| 24 | UGY16069 | Landrace | Uganda | NARO-NaCRRI |
| 25 | UGY16070 | Landrace | Uganda | NARO-NaCRRI |
| 26 | UGY16071 | Landrace | Uganda | NARO-NaCRRI |
| 27 | UGY16073 | Clone | Uganda | NARO-NaCRRI |
| 28 | UGY16074 | Landrace | Uganda | NARO-NaCRRI |
| 29 | UGY16076 | Landrace | Uganda | NARO-NaCRRI |
| 30 | UGY16091 | Clone | Uganda | NARO-NaCRRI |
| 31 | UGY19006 | Landrace | Uganda | NARO-NaCRRI |
| 32 | UGY19007 | Landrace | Uganda | NARO-NaCRRI |
| 33 | UGY19008 | Landrace | Uganda | NARO-NaCRRI |
| 34 | UGY19009 | Clone | Uganda | NARO-NaCRRI |
| 35 | UGY19013 | Landrace | Uganda | NARO-NaCRRI |
| 36 | UGY19014 | Landrace | Uganda | NARO-NaCRRI |
| 37 | UGY19015 | Landrace | Uganda | NARO-NaCRRI |
| 38 | UGY19016 | Landrace | Uganda | NARO-NaCRRI |
| 39 | UGY19017 | Landrace | Uganda | NARO-NaCRRI |
| 40 | UGY19018 | Landrace | Uganda | NARO-NaCRRI |
| 41 | UGY19019 | Landrace | Uganda | NARO-NaCRRI |
| 42 | UGY19020 | Landrace | Uganda | NARO-NaCRRI |
| 43 | UGY20013 | Clone | Ghana | CSIR-SARI |
| 44 | UGY20030 | Clone | Ghana | CSIR-SARI |
| 45 | UGY20031 | Clone | Nigeria | IITA/Ibadan |
| 46 | UGY20032 | Clone | Nigeria | IITA/Ibadan |
| 47 | UGY20033 | Landrace | Ghana | CSIR-SARI |
| 48 | UGY20034 | Clone | Ghana | CSIR-SARI |
| 49 | UGY20035 | Clone | Ghana | CSIR-SARI |
| 50 | UGY20036 | Clone | Ghana | CSIR-SARI |
| 51 | UGY20037 | Landrace | Ghana | CSIR-SARI |
| 52 | UGY20038 | Clone | Ghana | CSIR-SARI |
| 53 | UGY20039 | Landrace | Ghana | CSIR-SARI |
| 54 | UGY20040 | Clone | Ghana | CSIR-SARI |
| 55 | UGY20041 | Clone | Ghana | CSIR-SARI |
| 56 | UGY20042 | Landrace | Ghana | CSIR-SARI |
| 57 | UGY20043 | Clone | Ghana | CSIR-SARI |
| 58 | UGY20044 | Landrace | Ghana | CSIR-SARI |
| 59 | UGY20045 | Landrace | Ghana | CSIR-SARI |
| 60 | UGY20046 | Clone | Ghana | CSIR-SARI |
| 61 | UGY20047 | Clone | Ghana | CSIR-SARI |
| 62 | UGY20048 | Clone | Ghana | CSIR-SARI |
| 63 | UGY20050 | Landrace | Ghana | CSIR-SARI |
| 64 | UGY20051 | Landrace | Ghana | CSIR-SARI |
| 65 | UGY20052 | Landrace | Ghana | CSIR-SARI |
| 66 | UGY20053 | Clone | Ghana | CSIR-SARI |
| 67 | UGY20054 | Clone | Ghana | CSIR-SARI |
| 68 | UGY20055 | Landrace | Ghana | CSIR-SARI |
| 69 | UGY20056 | Clone | Ghana | CSIR-SARI |
| 70 | UGY20057 | Clone | Nigeria | IITA/Ibadan |
| 71 | UGY20058 | Clone | Nigeria | IITA/Ibadan |
| 72 | UGY20059 | Clone | Ghana | CSIR-SARI |
| 73 | UGY20060 | Clone | Ghana | CSIR-SARI |
| 74 | UGY20061 | Clone | Ghana | CSIR-SARI |
| 75 | UGY20062 | Clone | Ghana | CSIR-SARI |
| 76 | UGY20063 | Clone | Ghana | CSIR-SARI |
| 77 | UGY20064 | Clone | Ghana | CSIR-SARI |
| 78 | UGY20067 | Landrace | Ghana | CSIR-SARI |
| 79 | UGY20068 | Clone | Ghana | CSIR-SARI |
| 80 | UGY20069 | Landrace | Ghana | CSIR-SARI |
| 81 | UGY20070 | Clone | Ghana | CSIR-SARI |
| 82 | UGY20071 | Clone | Ghana | CSIR-SARI |
| 83 | UGY20072 | Landrace | Ghana | CSIR-SARI |
| 84 | UGY20073 | Clone | Ghana | CSIR-SARI |
| 85 | UGY20074 | Clone | Ghana | CSIR-SARI |
| 86 | UGY20075 | Landrace | Ghana | CSIR-SARI |
| 87 | UGY20076 | Landrace | Ghana | CSIR-SARI |
| 88 | UGY20077 | Clone | Ghana | CSIR-SARI |
| 89 | UGY20078 | Landrace | Ghana | CSIR-SARI |
| 90 | UGY20079 | Landrace | Ghana | CSIR-SARI |
| 91 | UGY20080 | Clone | Ghana | CSIR-SARI |
| 92 | UGY20081 | Clone | Ghana | CSIR-SARI |
| 93 | UGY20082 | Clone | Ghana | CSIR-SARI |
| 94 | UGY20083 | Clone | Ghana | CSIR-SARI |
| 95 | UGY20084 | Clone | Ghana | CSIR-SARI |
| 96 | UGY20085 | Landrace | Ghana | CSIR-SARI |
| 97 | UGY20086 | Clone | Ghana | CSIR-SARI |
| 98 | UGY20087 | Clone | Ghana | CSIR-SARI |
| 99 | UGY20088 | Landrace | Ghana | CSIR-SARI |
| 100 | UGY20089 | Landrace | Ghana | CSIR-SARI |
| 101 | UGY20090 | Landrace | Ghana | CSIR-SARI |
| 102 | UGY20091 | Clone | Ghana | CSIR-SARI |
| 103 | UGY20092 | Clone | Ghana | CSIR-SARI |
| 104 | UGY20093 | Clone | Ghana | CSIR-SARI |
| 105 | UGY20094 | Clone | Ghana | CSIR-SARI |
| 106 | UGY20095 | Clone | Ghana | CSIR-SARI |
| 107 | UGY20096 | Clone | Ghana | CSIR-SARI |
| 108 | UGY20097 | Clone | Ghana | CSIR-SARI |
| 109 | UGY20098 | Clone | Ghana | CSIR-SARI |
| 110 | UGY20099 | Clone | Ghana | CSIR-SARI |
| 111 | UGY20100 | Landrace | Ghana | CSIR-SARI |
| 112 | UGY20101 | Clone | Ghana | CSIR-SARI |
| 113 | UGY20102 | Clone | Ghana | CSIR-SARI |
| 114 | UGY20103 | Landrace | Ghana | CSIR-SARI |
| 115 | UGY20104 | Landrace | Ghana | CSIR-SARI |
| 116 | UGY20105 | Clone | Ghana | CSIR-SARI |
| 117 | UGY20106 | Landrace | Ghana | CSIR-SARI |
| 118 | UGY20107 | Landrace | Ghana | CSIR-SARI |
| 119 | UGY20108 | Landrace | Ghana | CSIR-SARI |
| 120 | UGY20109 | Landrace | Ghana | CSIR-SARI |
| 121 | UGY20110 | Clone | Ghana | CSIR-SARI |
| 122 | UGY20112 | Clone | Ghana | CSIR-SARI |
| 123 | UGY20113 | Clone | Ghana | CSIR-SARI |
| 124 | UGY20114 | Landrace | Ghana | CSIR-SARI |
| 125 | UGY20115 | Clone | Ghana | CSIR-SARI |
| 126 | UGY20116 | Clone | Nigeria | IITA/Ibadan |
| 127 | UGY20117 | Clone | Nigeria | IITA/Ibadan |
| 128 | UGY20118 | Clone | Nigeria | IITA/Ibadan |
| 129 | UGY20119 | Clone | Nigeria | IITA/Ibadan |
| 130 | UGY20120 | Clone | Nigeria | IITA/Ibadan |
| 131 | UGY20121 | Clone | Nigeria | IITA/Ibadan |
| 132 | UGY20122 | Clone | Nigeria | IITA/Ibadan |
| 133 | UGY20123 | Clone | Nigeria | IITA/Ibadan |
| 134 | UGY20124 | Clone | Nigeria | IITA/Ibadan |
| 135 | UGY20125 | Clone | Nigeria | IITA/Ibadan |
| 136 | UGY20126 | Clone | Nigeria | IITA/Ibadan |
| 137 | UGY20127 | Clone | Nigeria | IITA/Ibadan |
| 138 | UGY20128 | Clone | Nigeria | IITA/Ibadan |
| 139 | UGY20130 | Clone | Nigeria | IITA/Ibadan |
| 140 | UGY20131 | Clone | Nigeria | IITA/Ibadan |
| 141 | UGY20132 | Clone | Nigeria | IITA/Ibadan |
| 142 | UGY20133 | Clone | Nigeria | IITA/Ibadan |
| 143 | UGY20134 | Clone | Nigeria | IITA/Ibadan |
| 144 | UGY20135 | Clone | Nigeria | IITA/Ibadan |
| 145 | UGY20136 | Clone | Nigeria | IITA/Ibadan |
| 146 | UGY20137 | Clone | Nigeria | IITA/Ibadan |
| 147 | UGY20138 | Clone | Nigeria | IITA/Ibadan |
| 148 | UGY20139 | Clone | Nigeria | IITA/Ibadan |
| 149 | UGY20140 | Clone | Nigeria | IITA/Ibadan |
| 150 | UGY20141 | Clone | Nigeria | IITA/Ibadan |
| 151 | UGY20142 | Clone | Nigeria | IITA/Ibadan |
| 152 | UGY20143 | Clone | Nigeria | IITA/Ibadan |
| 153 | UGY20144 | Clone | Nigeria | IITA/Ibadan |
| 154 | UGY20145 | Clone | Nigeria | IITA/Ibadan |
| 155 | UGY20146 | Clone | Nigeria | IITA/Ibadan |
| 156 | UGY20147 | Clone | Nigeria | IITA/Ibadan |
| 157 | UGY20148 | Clone | Nigeria | IITA/Ibadan |
| 158 | UGY20149 | Clone | Nigeria | IITA/Ibadan |
| 159 | UGY20151 | Clone | Nigeria | IITA/Ibadan |
| 160 | UGY20152 | Clone | Nigeria | IITA/Ibadan |
| 161 | UGY20153 | Clone | Nigeria | IITA/Ibadan |
| 162 | UGY20154 | Clone | Nigeria | IITA/Ibadan |
| 163 | UGY20155 | Clone | Nigeria | IITA/Ibadan |
| 164 | UGY20156 | Clone | Nigeria | IITA/Ibadan |
| 165 | UGY20157 | Clone | Nigeria | IITA/Ibadan |
| 166 | UGY20158 | Clone | Nigeria | IITA/Ibadan |
| 167 | UGY20159 | Clone | Nigeria | IITA/Ibadan |
| 168 | UGY20160 | Clone | Nigeria | IITA/Ibadan |
| 169 | UGY20161 | Clone | Nigeria | IITA/Ibadan |
| 170 | UGY20162 | Clone | Nigeria | IITA/Ibadan |
| 171 | UGY20163 | Clone | Nigeria | IITA/Ibadan |
| 172 | UGY20165 | Clone | Nigeria | IITA/Ibadan |
| 173 | UGY20166 | Clone | Nigeria | IITA/Ibadan |
| 174 | UGY20167 | Clone | Nigeria | IITA/Ibadan |
| 175 | UGY20168 | Clone | Nigeria | IITA/Ibadan |
| 176 | UGY20169 | Clone | Nigeria | IITA/Ibadan |
| 177 | UGY20170 | Clone | Nigeria | IITA/Ibadan |
| 178 | UGY20171 | Clone | Nigeria | IITA/Ibadan |
| 179 | UGY20172 | Clone | Nigeria | IITA/Ibadan |
| 180 | UGY20173 | Clone | Nigeria | IITA/Ibadan |
| 181 | UGY20174 | Clone | Nigeria | IITA/Ibadan |
| 182 | UGY20175 | Clone | Nigeria | IITA/Ibadan |
| 183 | UGY20176 | Clone | Nigeria | IITA/Ibadan |
| 184 | UGY20177 | Clone | Nigeria | IITA/Ibadan |
| 185 | UGY20178 | Clone | Nigeria | IITA/Ibadan |
| 186 | UGY20179 | Clone | Nigeria | IITA/Ibadan |
| 187 | UGY20181 | Clone | Nigeria | IITA/Ibadan |
| 188 | UGY20182 | Clone | Nigeria | IITA/Ibadan |
| 189 | UGY20183 | Clone | Nigeria | IITA/Ibadan |
| 190 | UGY20184 | Clone | Nigeria | IITA/Ibadan |
| 191 | UGY20185 | Clone | Nigeria | IITA/Ibadan |
| 192 | UGY20186 | Clone | Nigeria | IITA/Ibadan |
| 193 | UGY20187 | Clone | Nigeria | IITA/Ibadan |
| 194 | UGY20188 | Clone | Nigeria | IITA/Ibadan |
| 195 | UGY20189 | Clone | Ghana | IITA/Ibadan |
| 196 | UGY20190 | Clone | Nigeria | IITA/Ibadan |
| 197 | UGY20191 | Clone | Nigeria | IITA/Ibadan |
| 198 | UGY20192 | Clone | Nigeria | IITA/Ibadan |
| 199 | UGY20193 | Clone | Nigeria | IITA/Ibadan |
| 200 | UGY20194 | Clone | Nigeria | IITA/Ibadan |
| 201 | UGY20195 | Clone | Ghana | CSIR-SARI |
| 202 | UGY20196 | Clone | Nigeria | IITA/Ibadan |
| 203 | UGY20199 | Clone | Nigeria | IITA/Ibadan |
| 204 | UGY20200 | Clone | Ghana | CSIR-SARI |
| 205 | UGY20201 | Clone | Ghana | CSIR-SARI |
| 206 | UGY20202 | Clone | Ghana | CSIR-SARI |
| 207 | UGY20203 | Clone | Ghana | CSIR-SARI |
