# Supplementary material for: Genetic diversity and population structure of Uganda’s yam (Dioscorea spp.) genetic resource based on DArTseq
Source: PLoS One. 2023 Feb 14;18(2):e0277537. doi: 10.1371/journal.pone.0277537 (PMC9928066; doi:10.1371/journal.pone.0277537)
Supplement: S2 Table — (DOCX) [file pone.0277537.s003.docx]

**S2 Table. Quality and summary statistics of DArTseq-SNPs on yam chromosomes**

| Linkage group (Chromosome) | Filtered SNPs | Chromosome Size (Mbs) | Minor allele frequency | Observed heterozygosity | Expected heterozygosity | Polymorphic information content | Call rate | Average Reproducibility |
| --- | --- | --- | --- | --- | --- | --- | --- | --- |
| 1 | 166 | 30.200 | 0.097 | 0.101 | 0.213 | 0.239 | 0.710 | 0.990 |
| 2 | 152 | 23.200 | 0.107 | 0.111 | 0.229 | 0.256 | 0.701 | 0.991 |
| 3 | 213 | 19.000 | 0.108 | 0.097 | 0.229 | 0.254 | 0.690 | 0.990 |
| 4 | 367 | 22.200 | 0.102 | 0.098 | 0.216 | 0.254 | 0.700 | 0.991 |
| 5 | 524 | 32.700 | 0.114 | 0.080 | 0.235 | 0.264 | 0.690 | 0.991 |
| 6 | 188 | 21.700 | 0.102 | 0.105 | 0.223 | 0.243 | 0.702 | 0.990 |
| 7 | 251 | 31.600 | 0.108 | 0.110 | 0.232 | 0.254 | 0.702 | 0.992 |
| 8 | 320 | 22.100 | 0.113 | 0.125 | 0.229 | 0.268 | 0.698 | 0.990 |
| 9 | 205 | 21.000 | 0.123 | 0.142 | 0.252 | 0.275 | 0.705 | 0.990 |
| 10 | 166 | 23.400 | 0.096 | 0.113 | 0.216 | 0.238 | 0.705 | 0.991 |
| 11 | 210 | 22.500 | 0.108 | 0.110 | 0.234 | 0.256 | 0.703 | 0.991 |
| 12 | 202 | 21.800 | 0.124 | 0.153 | 0.253 | 0.288 | 0.700 | 0.990 |
| 13 | 91 | 8.300 | 0.103 | 0.112 | 0.231 | 0.253 | 0.709 | 0.990 |
| 14 | 305 | 21.700 | 0.116 | 0.140 | 0.257 | 0.276 | 0.710 | 0.991 |
| 15 | 249 | 23.200 | 0.106 | 0.117 | 0.234 | 0.257 | 0.693 | 0.990 |
| 16 | 204 | 24.100 | 0.118 | 0.110 | 0.248 | 0.272 | 0.698 | 0.989 |
| 17 | 253 | 22.200 | 0.109 | 0.132 | 0.238 | 0.261 | 0.705 | 0.992 |
| 18 | 244 | 23.900 | 0.107 | 0.123 | 0.239 | 0.251 | 0.709 | 0.991 |
| 19 | 485 | 31.600 | 0.116 | 0.115 | 0.246 | 0.265 | 0.704 | 0.989 |
| 20 | 162 | 32.900 | 0.093 | 0.112 | 0.220 | 0.250 | 0.694 | 0.992 |
| Total/Average | 4957 | 479.300 | 0.109 | 0.115 | 0.234 | 0.259 | 0.701 | 0.991 |
