# Supplementary material for: Genetic diversity and population structure of Uganda’s yam (Dioscorea spp.) genetic resource based on DArTseq
Source: PLoS One. 2023 Feb 14;18(2):e0277537. doi: 10.1371/journal.pone.0277537 (PMC9928066; doi:10.1371/journal.pone.0277537)
Supplement: S3 Table — (DOCX) [file pone.0277537.s004.docx]

**S3 Table. List of genotypes, the proportion of grouping on based STRUCTURE analysis and geographical origin**

| Genotypes | ΔK = 2 | | ΔK = 3 | | | ΔK = 4 | | | | Geographical origin |
| --- | --- | --- | --- | --- | --- | --- | --- | --- | --- | --- |
|  | **POP I** | **POP II** | **POP II** | **POP III** | **POP I** | **POP I** | **POP II** | **POP III** | **POP IV** |  |
| UGY16002 | 1 | 0 | 0 | 0 | 1 | 1 | 0 | 0 | 0 | Uganda |
| UGY16003 | 1 | 0 | 0 | 0 | 1 | 1 | 0 | 0 | 0 | Uganda |
| UGY16004 | 0 | 1 | 1 | 0 | 0 | 0 | 0 | 1 | 0 | Ghana |
| UGY16006 | 1 | 0 | 0 | 0 | 1 | 1 | 0 | 0 | 0 | Uganda |
| UGY16010 | 0 | 1 | 1 | 0 | 0 | 0 | 0 | 1 | 0 | Uganda |
| UGY16011 | 1 | 0 | 0 | 0 | 1 | 1 | 0 | 0 | 0 | Uganda |
| UGY16013 | 1 | 0 | 0 | 0 | 1 | 1 | 0 | 0 | 0 | Uganda |
| UGY16016 | 1 | 0 | 0 | 0 | 1 | 1 | 0 | 0 | 0 | Uganda |
| UGY16022 | 1 | 0 | 0 | 0 | 1 | 1 | 0 | 0 | 0 | Uganda |
| UGY16026 | 1 | 0 | 0 | 0 | 1 | 1 | 0 | 0 | 0 | Uganda |
| UGY16027 | 1 | 0 | 0 | 0 | 1 | 1 | 0 | 0 | 0 | Uganda |
| UGY16029 | 1 | 0 | 0 | 0 | 1 | 1 | 0 | 0 | 0 | Uganda |
| UGY16033 | 1 | 0 | 0 | 0 | 1 | 1 | 0 | 0 | 0 | Uganda |
| UGY16035 | 0 | 1 | 1 | 0 | 0 | 0 | 0 | 1 | 0 | Uganda |
| UGY16037 | 1 | 0 | 0 | 0 | 1 | 1 | 0 | 0 | 0 | Uganda |
| UGY16039 | 1 | 0 | 0 | 0 | 1 | 1 | 0 | 0 | 0 | Uganda |
| UGY16041 | 1 | 0 | 0 | 0 | 1 | 1 | 0 | 0 | 0 | Uganda |
| UGY16042 | 0 | 1 | 1 | 0 | 0 | 0 | 0 | 1 | 0 | Uganda |
| UGY16045 | 1 | 0 | 0 | 0 | 1 | 1 | 0 | 0 | 0 | Uganda |
| UGY16064 | 1 | 0 | 0 | 1 | 0 | 0 | 1 | 0 | 0 | Uganda |
| UGY16065 | 1 | 0 | 0 | 1 | 0 | 0 | 1 | 0 | 0 | Uganda |
| UGY16066 | 1 | 0 | 0 | 1 | 0 | 0 | 1 | 0 | 0 | Uganda |
| UGY16068 | 1 | 0 | 0 | 0 | 1 | 1 | 0 | 0 | 0 | Uganda |
| UGY16069 | 1 | 0 | 0 | 0 | 1 | 1 | 0 | 0 | 0 | Uganda |
| UGY16070 | 1 | 0 | 0 | 1 | 0 | 0 | 1 | 0 | 0 | Uganda |
| UGY16071 | 1 | 0 | 0 | 0 | 1 | 1 | 0 | 0 | 0 | Uganda |
| UGY16073 | 1 | 0 | 0 | 1 | 0 | 0 | 1 | 0 | 0 | Uganda |
| UGY16074 | 1 | 0 | 0 | 0 | 1 | 1 | 0 | 0 | 0 | Uganda |
| UGY16076 | 1 | 0 | 0 | 1 | 0 | 0 | 1 | 0 | 0 | Uganda |
| UGY16091 | 1 | 0 | 0 | 1 | 0 | 0 | 1 | 0 | 0 | Uganda |
| UGY19006 | 1 | 0 | 0 | 1 | 0 | 0 | 1 | 0 | 0 | Uganda |
| UGY19007 | 1 | 0 | 0 | 0 | 1 | 1 | 0 | 0 | 0 | Uganda |
| UGY19008 | 1 | 0 | 0 | 0 | 1 | 1 | 0 | 0 | 0 | Uganda |
| UGY19009 | 1 | 0 | 0 | 1 | 0 | 0 | 1 | 0 | 0 | Uganda |
| UGY19013 | 1 | 0 | 0 | 0 | 1 | 1 | 0 | 0 | 0 | Uganda |
| UGY19014 | 1 | 0 | 0 | 0 | 1 | 1 | 0 | 0 | 0 | Uganda |
| UGY19015 | 1 | 0 | 0 | 0 | 1 | 1 | 0 | 0 | 0 | Uganda |
| UGY19016 | 1 | 0 | 0 | 0 | 1 | 1 | 0 | 0 | 0 | Uganda |
| UGY19017 | 1 | 0 | 0 | 0 | 1 | 1 | 0 | 0 | 0 | Uganda |
| UGY19018 | 1 | 0 | 0 | 0 | 1 | 1 | 0 | 0 | 0 | Uganda |
| UGY19019 | 1 | 0 | 0 | 0 | 1 | 1 | 0 | 0 | 0 | Uganda |
| UGY19020 | 0 | 1 | 1 | 0 | 0 | 0 | 0 | 1 | 0 | Uganda |
| UGY20013 | 1 | 0 | 0 | 0 | 1 | 1 | 0 | 0 | 0 | Ghana |
| UGY20030 | 0 | 1 | 1 | 0 | 0 | 0 | 0 | 1 | 0 | Ghana |
| UGY20031 | 0 | 1 | 1 | 0 | 0 | 0 | 0 | 1 | 0 | Nigeria |
| UGY20032 | 0 | 1 | 1 | 0 | 0 | 0 | 0 | 1 | 0 | Nigeria |
| UGY20033 | 1 | 0 | 0 | 1 | 0 | 0 | 1 | 0 | 0 | Ghana |
| UGY20034 | 1 | 0 | 0 | 1 | 0 | 0 | 1 | 0 | 0 | Ghana |
| UGY20035 | 1 | 0 | 0 | 1 | 0 | 0 | 1 | 0 | 0 | Ghana |
| UGY20036 | 1 | 0 | 0 | 1 | 0 | 0 | 1 | 0 | 0 | Uganda |
| UGY20037 | 1 | 0 | 0 | 0 | 1 | 1 | 0 | 0 | 0 | Ghana |
| UGY20038 | 1 | 0 | 0 | 1 | 0 | 0 | 1 | 0 | 0 | Ghana |
| UGY20039 | 1 | 0 | 0 | 1 | 0 | 0 | 1 | 0 | 0 | Ghana |
| UGY20040 | 1 | 0 | 0 | 1 | 0 | 0 | 1 | 0 | 0 | Ghana |
| UGY20041 | 1 | 0 | 0 | 1 | 0 | 0 | 1 | 0 | 0 | Ghana |
| UGY20042 | 1 | 0 | 0 | 1 | 0 | 0 | 1 | 0 | 0 | Ghana |
| UGY20043 | 1 | 0 | 0 | 1 | 0 | 0 | 1 | 0 | 0 | Ghana |
| UGY20044 | 1 | 0 | 0 | 1 | 0 | 0 | 1 | 0 | 0 | Ghana |
| UGY20045 | 1 | 0 | 0 | 1 | 0 | 0 | 1 | 0 | 0 | Ghana |
| UGY20046 | 0 | 1 | 1 | 0 | 0 | 0 | 0 | 1 | 0 | Ghana |
| UGY20047 | 1 | 0 | 0 | 0 | 1 | 1 | 0 | 0 | 0 | Ghana |
| UGY20048 | 1 | 0 | 0 | 1 | 0 | 0 | 1 | 0 | 0 | Ghana |
| UGY20050 | 1 | 0 | 0 | 1 | 0 | 0 | 1 | 0 | 0 | Ghana |
| UGY20051 | 1 | 0 | 0 | 1 | 0 | 0 | 0 | 0 | 1 | Ghana |
| UGY20052 | 1 | 0 | 0 | 1 | 0 | 0 | 1 | 0 | 0 | Ghana |
| UGY20053 | 1 | 0 | 0 | 1 | 0 | 0 | 1 | 0 | 0 | Ghana |
| UGY20054 | 1 | 0 | 0 | 0 | 1 | 1 | 0 | 0 | 0 | Ghana |
| UGY20055 | 1 | 0 | 0 | 0 | 1 | 1 | 0 | 0 | 0 | Ghana |
| UGY20056 | 1 | 0 | 0 | 1 | 0 | 0 | 1 | 0 | 0 | Ghana |
| UGY20057 | 1 | 0 | 0 | 1 | 0 | 0 | 0 | 0 | 1 | Nigeria |
| UGY20058 | 1 | 0 | 0 | 1 | 0 | 0 | 1 | 0 | 0 | Nigeria |
| UGY20059 | 1 | 0 | 0 | 1 | 0 | 0 | 1 | 0 | 0 | Ghana |
| UGY20060 | 1 | 0 | 0 | 1 | 0 | 0 | 1 | 0 | 0 | Ghana |
| UGY20061 | 1 | 0 | 0 | 1 | 0 | 0 | 1 | 0 | 0 | Ghana |
| UGY20062 | 1 | 0 | 0 | 0 | 1 | 1 | 0 | 0 | 0 | Ghana |
| UGY20063 | 1 | 0 | 0 | 0 | 1 | 1 | 0 | 0 | 0 | Ghana |
| UGY20064 | 1 | 0 | 0 | 1 | 0 | 0 | 1 | 0 | 0 | Ghana |
| UGY20067 | 1 | 0 | 0 | 1 | 0 | 0 | 1 | 0 | 0 | Ghana |
| UGY20068 | 1 | 0 | 0 | 1 | 0 | 0 | 1 | 0 | 0 | Ghana |
| UGY20069 | 1 | 0 | 0 | 0 | 1 | 1 | 0 | 0 | 0 | Ghana |
| UGY20070 | 1 | 0 | 0 | 1 | 0 | 0 | 1 | 0 | 0 | Ghana |
| UGY20071 | 1 | 0 | 0 | 1 | 0 | 0 | 1 | 0 | 0 | Ghana |
| UGY20072 | 1 | 0 | 0 | 0 | 1 | 1 | 0 | 0 | 0 | Ghana |
| UGY20073 | 1 | 0 | 0 | 1 | 0 | 0 | 1 | 0 | 0 | Ghana |
| UGY20074 | 1 | 0 | 0 | 1 | 0 | 0 | 1 | 0 | 0 | Ghana |
| UGY20075 | 1 | 0 | 0 | 1 | 0 | 0 | 1 | 0 | 0 | Ghana |
| UGY20076 | 1 | 0 | 0 | 0 | 1 | 1 | 0 | 0 | 0 | Ghana |
| UGY20077 | 1 | 0 | 0 | 0 | 1 | 1 | 0 | 0 | 0 | Ghana |
| UGY20078 | 1 | 0 | 0 | 1 | 0 | 0 | 1 | 0 | 0 | Ghana |
| UGY20079 | 1 | 0 | 0 | 1 | 0 | 0 | 1 | 0 | 0 | Ghana |
| UGY20080 | 1 | 0 | 0 | 0 | 1 | 1 | 0 | 0 | 0 | Ghana |
| UGY20081 | 1 | 0 | 0 | 0 | 1 | 1 | 0 | 0 | 0 | Ghana |
| UGY20082 | 1 | 0 | 0 | 1 | 0 | 0 | 1 | 0 | 0 | Ghana |
| UGY20083 | 1 | 0 | 0 | 1 | 0 | 0 | 1 | 0 | 0 | Ghana |
| UGY20084 | 1 | 0 | 0 | 1 | 0 | 0 | 1 | 0 | 0 | Ghana |
| UGY20085 | 1 | 0 | 0 | 1 | 0 | 0 | 1 | 0 | 0 | Ghana |
| UGY20086 | 1 | 0 | 0 | 1 | 0 | 0 | 1 | 0 | 0 | Ghana |
| UGY20087 | 1 | 0 | 0 | 1 | 0 | 0 | 1 | 0 | 0 | Ghana |
| UGY20088 | 1 | 0 | 0 | 1 | 0 | 0 | 1 | 0 | 0 | Ghana |
| UGY20089 | 1 | 0 | 0 | 1 | 0 | 0 | 1 | 0 | 0 | Ghana |
| UGY20090 | 1 | 0 | 0 | 1 | 0 | 0 | 1 | 0 | 0 | Ghana |
| UGY20091 | 1 | 0 | 0 | 0 | 1 | 1 | 0 | 0 | 0 | Ghana |
| UGY20092 | 1 | 0 | 0 | 1 | 0 | 0 | 1 | 0 | 0 | Ghana |
| UGY20093 | 1 | 0 | 0 | 1 | 0 | 0 | 1 | 0 | 0 | Ghana |
| UGY20094 | 1 | 0 | 0 | 1 | 0 | 0 | 1 | 0 | 0 | Ghana |
| UGY20095 | 1 | 0 | 0 | 1 | 0 | 0 | 1 | 0 | 0 | Ghana |
| UGY20096 | 1 | 0 | 0 | 1 | 0 | 0 | 1 | 0 | 0 | Ghana |
| UGY20097 | 1 | 0 | 0 | 1 | 0 | 0 | 1 | 0 | 0 | Ghana |
| UGY20098 | 1 | 0 | 0 | 1 | 0 | 0 | 1 | 0 | 0 | Ghana |
| UGY20099 | 1 | 0 | 0 | 1 | 0 | 0 | 1 | 0 | 0 | Ghana |
| UGY20100 | 1 | 0 | 0 | 1 | 0 | 0 | 1 | 0 | 0 | Ghana |
| UGY20101 | 1 | 0 | 0 | 1 | 0 | 0 | 1 | 0 | 0 | Ghana |
| UGY20102 | 1 | 0 | 0 | 0 | 1 | 1 | 0 | 0 | 0 | Ghana |
| UGY20103 | 1 | 0 | 0 | 1 | 0 | 0 | 0 | 0 | 1 | Ghana |
| UGY20104 | 1 | 0 | 0 | 1 | 0 | 0 | 1 | 0 | 0 | Ghana |
| UGY20105 | 1 | 0 | 0 | 1 | 0 | 0 | 1 | 0 | 0 | Ghana |
| UGY20106 | 1 | 0 | 0 | 1 | 0 | 0 | 1 | 0 | 0 | Ghana |
| UGY20107 | 1 | 0 | 0 | 1 | 0 | 0 | 1 | 0 | 0 | Ghana |
| UGY20108 | 1 | 0 | 0 | 1 | 0 | 0 | 1 | 0 | 0 | Ghana |
| UGY20109 | 1 | 0 | 0 | 0 | 1 | 1 | 0 | 0 | 0 | Ghana |
| UGY20110 | 1 | 0 | 0 | 0 | 1 | 1 | 0 | 0 | 0 | Ghana |
| UGY20112 | 1 | 0 | 0 | 1 | 0 | 0 | 1 | 0 | 0 | Ghana |
| UGY20113 | 1 | 0 | 0 | 0 | 1 | 1 | 0 | 0 | 0 | Ghana |
| UGY20114 | 1 | 0 | 0 | 0 | 1 | 1 | 0 | 0 | 0 | Ghana |
| UGY20115 | 0 | 1 | 1 | 0 | 0 | 0 | 0 | 1 | 0 | Ghana |
| UGY20116 | 1 | 0 | 0 | 1 | 0 | 0 | 1 | 0 | 0 | Nigeria |
| UGY20117 | 1 | 0 | 0 | 1 | 0 | 0 | 1 | 0 | 0 | Nigeria |
| UGY20118 | 1 | 0 | 0 | 1 | 0 | 0 | 1 | 0 | 0 | Nigeria |
| UGY20119 | 1 | 0 | 0 | 1 | 0 | 0 | 1 | 0 | 0 | Nigeria |
| UGY20120 | 1 | 0 | 0 | 1 | 0 | 0 | 1 | 0 | 0 | Nigeria |
| UGY20121 | 1 | 0 | 0 | 1 | 0 | 0 | 1 | 0 | 0 | Nigeria |
| UGY20122 | 1 | 0 | 0 | 1 | 0 | 0 | 1 | 0 | 0 | Nigeria |
| UGY20123 | 1 | 0 | 0 | 0 | 1 | 1 | 0 | 0 | 0 | Nigeria |
| UGY20124 | 1 | 0 | 0 | 1 | 0 | 0 | 1 | 0 | 0 | Nigeria |
| UGY20125 | 1 | 0 | 0 | 1 | 0 | 0 | 1 | 0 | 0 | Nigeria |
| UGY20126 | 1 | 0 | 0 | 1 | 0 | 0 | 1 | 0 | 0 | Nigeria |
| UGY20127 | 1 | 0 | 0 | 0 | 1 | 1 | 0 | 0 | 0 | Nigeria |
| UGY20128 | 1 | 0 | 0 | 1 | 0 | 0 | 1 | 0 | 0 | Nigeria |
| UGY20130 | 1 | 0 | 0 | 1 | 0 | 0 | 1 | 0 | 0 | Nigeria |
| UGY20131 | 1 | 0 | 0 | 1 | 0 | 0 | 0 | 0 | 1 | Nigeria |
| UGY20132 | 1 | 0 | 0 | 1 | 0 | 0 | 1 | 0 | 0 | Nigeria |
| UGY20133 | 1 | 0 | 0 | 1 | 0 | 0 | 1 | 0 | 0 | Nigeria |
| UGY20134 | 1 | 0 | 0 | 1 | 0 | 0 | 1 | 0 | 0 | Nigeria |
| UGY20135 | 1 | 0 | 0 | 1 | 0 | 0 | 1 | 0 | 0 | Nigeria |
| UGY20136 | 1 | 0 | 0 | 1 | 0 | 0 | 1 | 0 | 0 | Nigeria |
| UGY20137 | 1 | 0 | 0 | 1 | 0 | 0 | 1 | 0 | 0 | Nigeria |
| UGY20138 | 1 | 0 | 0 | 0 | 1 | 1 | 0 | 0 | 0 | Nigeria |
| UGY20139 | 1 | 0 | 0 | 0 | 1 | 1 | 0 | 0 | 0 | Nigeria |
| UGY20140 | 1 | 0 | 0 | 1 | 0 | 0 | 0 | 0 | 1 | Nigeria |
| UGY20141 | 1 | 0 | 0 | 0 | 1 | 1 | 0 | 0 | 0 | Nigeria |
| UGY20142 | 1 | 0 | 0 | 1 | 0 | 0 | 1 | 0 | 0 | Nigeria |
| UGY20143 | 1 | 0 | 0 | 1 | 0 | 0 | 1 | 0 | 0 | Nigeria |
| UGY20144 | 1 | 0 | 0 | 1 | 0 | 0 | 1 | 0 | 0 | Nigeria |
| UGY20145 | 1 | 0 | 0 | 0 | 1 | 1 | 0 | 0 | 0 | Nigeria |
| UGY20146 | 1 | 0 | 0 | 1 | 0 | 0 | 1 | 0 | 0 | Nigeria |
| UGY20147 | 1 | 0 | 0 | 1 | 0 | 0 | 1 | 0 | 0 | Nigeria |
| UGY20148 | 1 | 0 | 0 | 1 | 0 | 0 | 1 | 0 | 0 | Nigeria |
| UGY20149 | 1 | 0 | 0 | 0 | 1 | 1 | 0 | 0 | 0 | Nigeria |
| UGY20151 | 1 | 0 | 0 | 1 | 0 | 0 | 1 | 0 | 0 | Nigeria |
| UGY20152 | 1 | 0 | 0 | 1 | 0 | 0 | 1 | 0 | 0 | Nigeria |
| UGY20153 | 1 | 0 | 0 | 1 | 0 | 0 | 1 | 0 | 0 | Nigeria |
| UGY20154 | 1 | 0 | 0 | 1 | 0 | 0 | 1 | 0 | 0 | Nigeria |
| UGY20155 | 1 | 0 | 0 | 1 | 0 | 0 | 0 | 0 | 1 | Nigeria |
| UGY20156 | 1 | 0 | 0 | 1 | 0 | 0 | 1 | 0 | 0 | Nigeria |
| UGY20157 | 1 | 0 | 0 | 0 | 1 | 1 | 0 | 0 | 0 | Nigeria |
| UGY20158 | 1 | 0 | 0 | 1 | 0 | 0 | 1 | 0 | 0 | Nigeria |
| UGY20159 | 1 | 0 | 0 | 1 | 0 | 0 | 1 | 0 | 0 | Nigeria |
| UGY20160 | 1 | 0 | 0 | 1 | 0 | 0 | 1 | 0 | 0 | Nigeria |
| UGY20161 | 1 | 0 | 0 | 1 | 0 | 0 | 1 | 0 | 0 | Nigeria |
| UGY20162 | 1 | 0 | 0 | 0 | 1 | 0 | 1 | 0 | 0 | Nigeria |
| UGY20163 | 0 | 1 | 1 | 0 | 0 | 0 | 0 | 1 | 0 | Nigeria |
| UGY20165 | 1 | 0 | 0 | 0 | 1 | 1 | 0 | 0 | 0 | Nigeria |
| UGY20166 | 1 | 0 | 0 | 0 | 1 | 1 | 0 | 0 | 0 | Nigeria |
| UGY20167 | 1 | 0 | 0 | 1 | 0 | 0 | 1 | 0 | 0 | Nigeria |
| UGY20168 | 1 | 0 | 0 | 1 | 0 | 0 | 1 | 0 | 0 | Nigeria |
| UGY20169 | 1 | 0 | 0 | 1 | 0 | 0 | 1 | 0 | 0 | Nigeria |
| UGY20170 | 1 | 0 | 0 | 1 | 0 | 0 | 1 | 0 | 0 | Nigeria |
| UGY20171 | 1 | 0 | 0 | 1 | 0 | 0 | 1 | 0 | 0 | Nigeria |
| UGY20172 | 1 | 0 | 0 | 1 | 0 | 0 | 1 | 0 | 0 | Nigeria |
| UGY20173 | 1 | 0 | 0 | 1 | 0 | 0 | 1 | 0 | 0 | Nigeria |
| UGY20174 | 1 | 0 | 0 | 1 | 0 | 0 | 1 | 0 | 0 | Nigeria |
| UGY20175 | 1 | 0 | 0 | 1 | 0 | 0 | 1 | 0 | 0 | Nigeria |
| UGY20176 | 1 | 0 | 0 | 0 | 1 | 1 | 0 | 0 | 0 | Nigeria |
| UGY20177 | 1 | 0 | 0 | 1 | 0 | 0 | 1 | 0 | 0 | Nigeria |
| UGY20178 | 1 | 0 | 0 | 1 | 0 | 0 | 1 | 0 | 0 | Nigeria |
| UGY20179 | 1 | 0 | 0 | 1 | 0 | 0 | 1 | 0 | 0 | Nigeria |
| UGY20181 | 1 | 0 | 0 | 0 | 1 | 1 | 0 | 0 | 0 | Nigeria |
| UGY20182 | 1 | 0 | 0 | 1 | 0 | 0 | 1 | 0 | 0 | Nigeria |
| UGY20183 | 1 | 0 | 0 | 1 | 0 | 0 | 1 | 0 | 0 | Nigeria |
| UGY20184 | 1 | 0 | 0 | 1 | 0 | 0 | 1 | 0 | 0 | Nigeria |
| UGY20185 | 1 | 0 | 0 | 1 | 0 | 0 | 1 | 0 | 0 | Nigeria |
| UGY20186 | 1 | 0 | 0 | 1 | 0 | 0 | 1 | 0 | 0 | Nigeria |
| UGY20187 | 1 | 0 | 0 | 1 | 0 | 0 | 1 | 0 | 0 | Nigeria |
| UGY20188 | 1 | 0 | 0 | 1 | 0 | 0 | 0 | 0 | 1 | Nigeria |
| UGY20189 | 1 | 0 | 0 | 1 | 0 | 0 | 1 | 0 | 0 | Ghana |
| UGY20190 | 1 | 0 | 0 | 1 | 0 | 0 | 1 | 0 | 0 | Nigeria |
| UGY20191 | 0 | 1 | 1 | 0 | 0 | 0 | 0 | 1 | 0 | Nigeria |
| UGY20192 | 1 | 0 | 0 | 1 | 0 | 0 | 1 | 0 | 0 | Nigeria |
| UGY20193 | 1 | 0 | 0 | 1 | 0 | 0 | 1 | 0 | 0 | Nigeria |
| UGY20194 | 1 | 0 | 0 | 1 | 0 | 0 | 1 | 0 | 0 | Nigeria |
| UGY20195 | 1 | 0 | 0 | 1 | 0 | 0 | 0 | 0 | 1 | Ghana |
| UGY20196 | 1 | 0 | 0 | 1 | 0 | 0 | 1 | 0 | 0 | Nigeria |
| UGY20199 | 1 | 0 | 0 | 0 | 1 | 1 | 0 | 0 | 0 | Nigeria |
| UGY20200 | 1 | 0 | 0 | 1 | 0 | 0 | 1 | 0 | 0 | Ghana |
| UGY20201 | 1 | 0 | 0 | 1 | 0 | 0 | 1 | 0 | 0 | Ghana |
| UGY20202 | 1 | 0 | 0 | 1 | 0 | 0 | 1 | 0 | 0 | Ghana |
| UGY20203 | 1 | 0 | 0 | 1 | 0 | 0 | 1 | 0 | 0 | Ghana |
| Proportion | 0.94 | 0.06 | 0.06 | 0.662 | 0.278 | 0.28 | 0.548 | 0.06 | 0.112 |  |
