# Supplementary material for: Genetic diversity and population structure of Uganda’s yam (Dioscorea spp.) genetic resource based on DArTseq
Source: PLoS One. 2023 Feb 14;18(2):e0277537. doi: 10.1371/journal.pone.0277537 (PMC9928066; doi:10.1371/journal.pone.0277537)
Supplement: Attachment — Submitted filename: PONE-D-22-02542_Reviewers comments Addressed.docx [file pone.0277537.s007.docx]

12^th^ October 2022

Academic Editor

PLOS ONE

Dear Editor,

I respectfully write to thank the reviewers for their valuable comments.

Kindly find below the actions taken on the reviewers’ comments for the manuscript “**PONE-D-22-02542 - Genetic diversity and population structure of Uganda’s yam (***Dioscorea spp***.) genetic resource based on DArTseq”**

| **Reviewer One** | | | |
| --- | --- | --- | --- |
| **No.** | **Reviewers’ comments** | **Action** | **Page/line** |
| 1 | Cite this paper: Bredeson, J.V., Lyons, J.B., Oniyinde, I.O. et al. Chromosome evolution and the genetic basis of agronomically important traits in greater yam. Nat Commun 13, 2001 (2022) | Duly cited | Page 3 Lines 55 – 57  Page 16 Lines 344 - 346 |
| 2 | Line 69-75, is too long a sentence. Just present the results in short sentences, which is easy to read and understanding. Please re-write this!! | Modified as suggested |  |
| 3 | In the Materials & methods, DArTseq marker genotyping is explained, but it seems that this part was done by Diversity Array Technology Inc, Canberra, Australia. If so, it should be clearly noted and cited in Materials & Methods. See these example papers of different plant species: Hassani, et al., 2020) and Mahboubi, et al., 2020). | Citations duly added | Page 6 Line 126 |
| 4 | Also its recommended to cite some research paper for the application of DArTseq markers on different plant species and then cite the paper of Agre et al. (2019). | Duly cited throughout the manuscript |  |
| 5 | In Table 1, it’s better to add some more information about the markers coverage on each chromosomes and also the amount of mean and range of PIC for each chromosome (see example paper : (Farahani et al. 2019; Hassani et al. 2020). | Table 1 updated and presented as S1 table | S1 table |
| 6 | Line 244-250, should be removed to Introduction part. | Moved accordingly | Page 4 lines 69 - 73 |
| 7 | Line 278-294: This is just the repetition of results. Here authors should more focus on the application of these generated data for any future germplasm conservation and breeding programs in Yam. For example, there were no information about the certain genotypes that are very diverse as a hetrotic groups that can used in breeding programs or mentioned genotypes/sources from very close sources, if their morphological characteristics (if available) are the same or difference!!!. These part should be more discussed. | The session duly updated as suggested |  |
| 8 | An table for markers information like name, chromosome, physical position, PIC and some quality parameters like: call rate and reproducibility should be presented as supplementary Table | Moved accordingly | S1 table |
| 9 | Text need to extensive English grammar check. |  |  |
| **Reviewer two** | | | |
| 1 | The authors did not express their aim well enough in the last paragraph of the introduction section and should be written more clearly about how the results of the study will contribute to the literature. | Aim of study restructured | Page 5 lines 102 - 110 |
| 2 | L92.P4.The authors stated that “genotypes sourced from three different geographical origin” in the abstract file. Please write these origins in detail by specifying the genotype number. | Detailed numbers in S1 Table |  |
| 3 | L94.P4. The S1 provided the genotypes ID, description, and geographical origin. Please give more detail about passport data of plant materials; Collection Site, District, Village, Altitude (m), and Coordinates | Table updated with institution sourced from. Unfortunately, detailed information such as district, and village were not captured about the genotypes because they are not available. |  |
| 4 | L98.P5. Please give more detail about the growing condition before the DNA extraction. | Updated accordingly | Page 6 lines 115 - 116 |
| 5 | L98.P5. Please give more detail about the DNA extraction protocol. | Updated accordingly | Page 6 lines 116 - 119 |
| 6 | L103.P8. Please give more detailed information about how minor allele frequency, observed heterozygosity, expected heterozygosity, and gene diversity were determined in the statistical analysis section. | Update accordingly | Page 7 line 147 - 160 |
| 7 | Please provide all figures with 300dpi resolution. | Figures uploaded onto PACE and met PLOS requirements |  |
| 8 | L233. P11. Please revise as “..mixture of all genotype sources from..” instead of “mixture of all genotype’s sources from” | Update accordingly |  |
| 9 | L246-250.P12. Please also provide these literatures in the introduction section. | Update accordingly |  |
| 10 | L262. P12. To support your statements, add a more recent study for common bean; Nadeem et al. (2018) | Update accordingly |  |
| 11 | The discussion section should be improved. The conclusion should be shortened and concrete. The reference section should be double-checked for the journal formatting rules | Update accordingly |  |

Thank you for your consideration of this manuscript.

Sincerely

Emmanuel Amponsah Adjei (Ph. D Student)

Department of Agricultural Production

Makerere University

P. O. Box 7062

Kampala - Uganda
